# Post-Harvest UV-C Treatment of Microgreens for Inactivation of *Salmonella enterica*, *Escherichia coli* O157:H7 and *Listeria monocytogenes*

**DOI:** 10.3390/foods15060974

**Published:** 2026-03-10

**Authors:** Sefa Işık, Bülent Çetin, Juan Moreira, Zeynal Topalcengiz

**Affiliations:** 1 Department of Food Processing, Vocational School of Technical Sciences, Muş Alparslan University, 49250 Muş, Türkiye; s.isik@alparslan.edu.tr; 2 Department of Food Engineering, Faculty of Agriculture, Atatürk University, 25240 Erzurum, Türkiye; bcetin@atauni.edu.tr; 3 Department of Food Science and Human Nutrition, College of Health and Human Sciences, Colorado State University, Fort Collins, CO 80526, USA; juan.moreiracalix@colostate.edu; 4 Department of Food Engineering, Faculty of Engineering and Architecture, Muş Alparslan University, 49250 Muş, Türkiye

**Keywords:** microgreens, UV-C treatment, foodborne pathogens, post-harvest microbial control, refrigerated storage

## Abstract

There is a high risk of transfer of foodborne pathogens to the edible part of microgreens when seeds, irrigation water or soilless substrates are contaminated. Post-harvest sanitizer treatments are generally not preferred due to the fragility of microgreens. In this study, the effectiveness of post-harvest UV-C treatment was evaluated against *Salmonella enterica*, Shiga toxin-producing *Escherichia coli* O157:H7, and *Listeria monocytogenes* in sunflower and radish microgreens. Agricultural perlite soaked with plant nutrient solution was artificially contaminated with foodborne pathogens at a concentration of 10^5^–10^6^ CFU/g to serve as the soilless substrate. UV-C was applied to harvested microgreens uni- and bidirectionally with doubled exposure at varying distances (10, 20, and 30 cm) and exposure times (5, 10, 20, 30, 60, and 120 s). UV-C doses ranged from 0.03 to 2.07 kJ/m^2^, depending on treatment distance and exposure time. The survival of pathogens in treated microgreens was also determined at 4 °C for 14 days. The highest pathogen inhibition was achieved with bidirectional UV-C treatment at a 10 cm distance for 120 s (*p* < 0.05), yielding reductions of up to 3.1, 3.0, and 2.0 log CFU/g for *S. enterica*, *E. coli* O157:H7, and *L. monocytogenes*, respectively. Pathogen inhibition decreased significantly with increasing distance (*p* < 0.05). During subsequent refrigerated storage after UV-C treatment, pathogen populations increased by 0.3–1.7 log CFU/g. These results demonstrate that UV-C treatment can significantly reduce pathogen populations on microgreens as a post-harvest treatment strategy but cannot fully address food safety concerns about these immature seedlings.

## 1. Introduction

Consumer interest in and demand for microgreens has been increasing in recent years, mainly due to their health-promoting rich nutritional content and functional effects [1,2,3], their desired sensory properties as ingredients in various cuisines [4], and the relatively easy and inexpensive conditions required for growing them in indoor setups such as homes or small commercial facilities. Although the same food safety standards apply to microgreens as to fully grown produce in the United States [5], the transfer of foodborne pathogens to the edible part of seedlings at high concentrations from several sources during growing and harvesting has been reported for twenty types of microgreens [6]. Recent recalls in North America due to the occurrence of foodborne pathogens such as *Salmonella* spp. and *Listeria monocytogenes* have drawn the attention of researchers and authorities to the safety of these small seedlings [7,8].

Major microgreen contamination sources include contaminated seeds, growing media, irrigation water, nutrient solution, workers with poor health or sanitation, and equipment and buildings [6,8,9]. Therefore, prevention of bacterial and viral contamination of microgreens at the pre-harvest level is essential for addressing safety concerns [6,8,10,11,12,13,14,15]. Once contamination occurs at the pre-harvest level, reducing foodborne pathogens on microgreens through thermal and chemical treatments is not considered a viable option by growers due to the fragile structure of the plant surface, the small size of the leaves, and the short storage periods affecting sensory quality.

Non-thermal preservation techniques have been shown to be effective in maintaining the nutritional value and sensory quality of foods while also playing a vital role in the inactivation of both pathogenic and spoilage (saprophytic) microorganisms [16]. Ultraviolet-C (UV-C) treatment, within the 100–280 nanometer wavelength range, achieves microbial inactivation by disrupting DNA transcription and replication and inducing photochemical changes in nucleic acids and cell membrane proteins [17,18]. Specifically, UV-C light at a wavelength of 254 nanometers has been reported to exhibit the highest microbial inactivation efficacy and is widely applied to reduce microbial loads and inhibit pathogens on surfaces of fruits and vegetables, with acceptable or no visible defects on crop surfaces, as a chemical-free technique [19,20,21,22,23,24]. The efficacy of UV-C treatment for elimination of microbial contamination or load depends on various factors related to treatment conditions and time, the type and state of microorganisms, and the surface characteristics of the produce [25]. Despite ongoing research efforts, UV-C treatment often provides limited microbial reduction on some fruit and vegetable surfaces since the influencing factors cannot be fully standardized in most practical applications.

The fragile structure of microgreens limits the number of possible post-harvest safety interventions and mitigation strategies applicable to this type of produce. Therefore, there is an urgent need to evaluate non-thermal and minimally invasive methods that can mitigate risk of pathogen occurrence on microgreens. Such methods must ensure the safety of foods without diminishing their visual appeal, freshness, and sensory quality, especially in cases where possible contamination may happen during pre-harvest-level activities and harvesting. In the present study, the efficacy of UV-C (253.7 nm) treatment was evaluated for radish and sunflower microgreens. UV-C was applied at different distances (10, 20, and 30 cm), directions (unidirectional and bidirectional), and durations (5, 10, 20, 30, 60, and 120 s) against *S. enterica*, *E. coli* O157:H7, and *L. monocytogenes*. Furthermore, changes in pathogen populations were observed on microgreens stored under refrigerated conditions (4 °C) after UV-C treatment.

## 2. Materials and Methods

### 2.1. Microgreens and Growth Media

Radish (Cherry Belle) and sunflower microgreens were selected for their relatively large size and greater durability under UV-C treatment. The microgreen seeds were obtained from certified local suppliers and were guaranteed to be free of chemicals and other preservatives. Agricultural perlite (Mita Tarım, Antalya, Türkiye), with a particle size of 3–6 mm and a pH range of 6.5–7.5, was used as the soilless substrate. The perlite was sterilized at 121 °C for 15 min prior to use. The plant nutrient solution was prepared by mixing 2 mL of solutions A and B in 1 L of sterile distilled water, in accordance with the manufacturer’s instructions. The chemical composition of each solution was described previously in Işık et al. [12].

### 2.2. Bacterial Strains and Inoculum Preparation

Three strains of ampicillin-resistant *Salmonella enterica* [serovar Enteritidis (ATCC 13076), Infantis (food isolate), and serovar Typhimurium (ATCC 14028)] and *E. coli* O157:H7 [ZT9 (food isolate), ZT10 (food isolate), and ATCC 35150] were used in the present study. A modified version of the protocol described by Parnell et al. [26] was employed to make pathogen strains resistant to ampicillin. Ampicillin was utilized at a concentration of 120 µL/mL (Sigma-Aldrich, Steinheim, Germany). Three strains of *L. monocytogenes* [serotype 1/2a (ATCC 51774), serotype 1/2b (ATCC 7644), and serotype 4b (ATCC 13932)] were included in the study.

The strains of *Salmonella* and *E. coli* O157:H7, initially stored at −80 °C, were activated by streaking them onto tryptic soy agar supplemented with 100 μg/mL ampicillin (TSA, Biolife, Milan, Italy). The plates were incubated at 35 ± 2 °C for 24 ± 2 h. A single colony from the activated cultures was inoculated into 10 mL of tryptic soy broth (TSB, Merck KGaA, Darmstadt, Germany) supplemented with 100 μg/mL ampicillin (Sigma-Aldrich, St. Louis, MO, USA). Following an incubation period of 20 ± 1 h at 35 ± 2 °C, 100 μL of the culture was transferred to a sterile 15 mL centrifuge tube (Labsolute, Renningen, Germany) containing 10 mL of TSB with 100 μg/mL ampicillin. Subsequently, the culture was incubated at 35 ± 2 °C for an additional 20 ± 1 h.

The same procedure was employed for the strains of *L. monocytogenes* in TSB supplemented with 0.6% yeast extract (Biolife, Milan, Italy). After the incubation period, the cultures were centrifuged at 5300× *g* for 10 min (Thermo Scientific Labofuge 200 Benchtop Centrifuge, Osterode, Germany). The supernatant was discarded, and the cell pellets were washed twice with 10 mL of sterile 0.1% peptone solution (Biolife, Milan, Italy). Following the washing steps, 5 mL of sterile peptone solution was added to the pellet, yielding a bacterial suspension with a cell density of 10^8^ to 10^9^ CFU/mL. This suspension was diluted to have an inoculum with a concentration of 10^7^–10^8^ CFU/g for subsequent inoculation.

### 2.3. Inoculation of Perlite

Prior to inoculation, 250 mL of plant nutrient solution was added to 750 mL of perlite in stomacher bags (BagLight^®^, Interscience, Saint Nom, France) to produce growth medium for microgreens. The plant nutrition-soaked perlite was then inoculated by adding 10 mL of separate pathogen inoculums to the stomacher bags, resulting in a target concentration level of 10^5^–10^6^ CFU/g. Three strain cocktails of each pathogen were inoculated in sterile perlite separately. The stomacher bags were then agitated for two minutes to create a homogeneous mixture. Approximately 500 mL of the inoculated perlite of each pathogen was placed with even thickness into uncapped black plastic containers (1000 mL) used as growing trays. Seeds were distributed across the surface of the perlite and covered with the remaining inoculated perlite (~250 mL) at around ~1 cm thickness as detailed by Işık et al. [27].

### 2.4. Growing and Harvesting Microgreens

The inoculated seeded containers, previously soaked in plant nutrition-soaked perlite were kept in the dark and spray-irrigated for three days. On day 4, the microgreens were placed under white fluorescent light with an intensity of approximately 400 µmol/m^2^ (C.E.M. DT-1309 Luxmeter, Shenzhen, China), following a photoperiod of 12 h of light and 12 h of darkness until harvest. Microgreens were watered by pumping plant nutrient solution with the help of 50 mL sterile syringes. Until harvest day, the temperature and humidity of the growth room were monitored with a data logger (Loyka Instruments, Istanbul, Türkiye).

The microgreens were harvested once the true leaves had developed, which occurred after 9 ± 1 days for sunflower and 8 ± 1 days for radish. For each sample, approximately 5 g of microgreens were harvested from a height of approximately 1 cm above the perlite surface using sterile scissors [27]. The harvested microgreens were immediately placed on clamshells for the UV-C treatments.

### 2.5. UV-C Treatment

**Figure 1:**
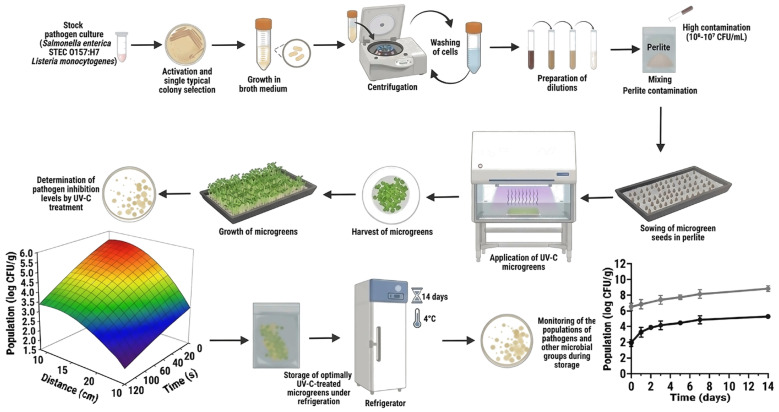
A schematic diagram illustrating the UV-C treatment setup and storage.

A UV-C lamp emitting at a wavelength of 253.7 nm was used for the treatments. Prior to the experiments, the biosafety cabinet was sterilized and stabilized by exposure to UV-C for 30 min. Microgreen samples (5 g) were placed on round clamshells (17 cm diameter and 2 cm depth), which had been sterilized under UV-C for 10 min before use. The treatment parameters included three distances (10, 20, and 35 cm), six exposure times (5, 10, 20, 30, 60, and 120 s), and two application strategies: unidirectional (one-sided exposure) and bidirectional (two-sided exposure). For the bidirectional application, samples were turned over using a second sterile plate after the first exposure and subjected to an equivalent additional dose, resulting in a total UV-C dose twice that of the unidirectional treatment. A schematic diagram illustrating the UV-C treatment setup and storage is shown in Figure 1.

Treatment intensity and dose were calculated from the technical data of the UV-C lamp used. The UV-C intensity (Equation (1)) was determined by dividing the total light power (watt, W) coming from all directions on the surface of the target area by the surface area of the target area. The UV-C dose (Equation (2)) was determined by dividing the total light energy (joule, J) coming from all directions on the target area by the surface area of the target area [28], which varied according to times and distances, as shown in Table 1. The study was carried out using four biological replicates (*n* = 4).

(1) UV-C Intensity (W/m^2^) = Lamp power (W) × Intensity factor (1/m^2^)

(2) UV-C Dose (kj/m^2^) = UV-C Intensity (W/m^2^) × Treatment time (s)

### 2.6. Microbiological Analysis

Following the UV-C treatments, microgreen samples (5 g) on clamshells were placed in sterile stomacher bags with 45 mL peptone water and stomached for 2 min (CLS, CLPM-400, Ankara, Türkiye). *Salmonella* populations were enumerated by spread plating on xylose lysine deoxycholate agar (XLDA, Oxoid, Hants, UK) supplemented with 100 µg/mL ampicillin. *E. coli* O157:H7 was determined on sorbitol MacConkey agar with cefixime–tellurite supplement (SMAC-CT-A, Condalab, Madrid, Spain; CT Supplement, Oxoid, Hants, UK) and 100 µg/mL ampicillin. *L. monocytogenes* was enumerated on PALCAM agar with PAC supplement (Neogen, Heywood, UK). All plates were incubated at 35 ± 2 °C for 24–48 h until the observation of typical colonies, depending on the growth rate of the target pathogen. The population of total mesophilic aerobic bacteria (TMAB) was enumerated on tryptic soy agar (TSA, Biolife; Milan, Italy) after incubation for 48 ± 2 h at 35 ± 2 °C. Total yeast and mold counts (TYMC) were determined on potato dextrose agar (PDA, Condalab, Madrid, Spain) after incubation at 25 °C for 3–5 days. Throughout the study, TMAB and TYMC populations were monitored using microgreens artificially contaminated with *E. coli* O157:H7. Prior to the study, all seeds were examined for the presence of *E. coli* O157:H7, *S. enterica*, and *L. monocytogenes* in the background microbiota by plating them on selective agars.

### 2.7. Post-UV-C Storage and Microbial Evaluation

Upon visual examination, sunflower microgreens exposed to UV-C treatment for 120 s at 10 cm appeared to not present significant changes in their appearance, whereas radish microgreens exhibited wilting and an undesirable cooked odor under the same conditions. Therefore, microgreens treated with the UV-C dose that preserved appealing shape and color while providing the highest pathogen inhibition (60 s dual-side exposure at 10 cm) were selected for a further 14 days of storage to observe the microbiological quality of microgreens refrigerated at 4 ± 2 °C. Microgreens (5 g) were stored in square clamshells (11 × 11 cm side length and 2.5 cm depth), which had been sterilized under UV-C for 10 min before use. A separate set of treated microgreen samples were tested on days 0, 1, 3, 5, 7 and 14 to determine the population of *E. coli* O157:H7, *S. enterica*, *L. monocytogenes,* TMAB, and TYMC as described above. The study was conducted in parallel with three independent replications.

### 2.8. Statistical Analysis

The data were subjected to one-way analysis of variance (ANOVA) in SPSS for Windows version 26 (IBM Corp., Armonk, NY, USA) and statistically compared by Tukey’s HSD test. Three-dimensional graphs were produced using SigmaPlot 10.0 software (Dundas Software Inc., Toronto, Canada).

## 3. Results

### 3.1. Transfer of Pathogens to Microgreens

The concentrations of the transferred populations of *Salmonella*, *E. coli* O157:H7, and *L. monocytogenes* on radish microgreens grown in contaminated plant nutrient solution-soaked perlite were 5.8 ± 0.2, 7.2 ± 0.2, and 5.4 ± 0.1 log CFU/g, respectively, before UV-C exposure. For sunflower microgreens, the concentrations of the corresponding pre-exposure pathogen populations were 4.5 ± 0.3, 6.9 ± 0.2, and 3.3 ± 0.2 log CFU/g. The concentrations of the TMAB and TYMC populations in radish microgreens were 7.6 ± 0.1 log CFU/g and 3.1 ± 0.4 log CFU/g, respectively. Similarly, the values for sunflower microgreens were 6.8 ± 0.1 log CFU/g (TMAB) and 3.0 ± 0.7 log CFU/g (TYMC).

### 3.2. Inhibition of S. enterica, E. coli O157:H7 and L. monocytogenes by UV-C Treatment

**Figure 2:**
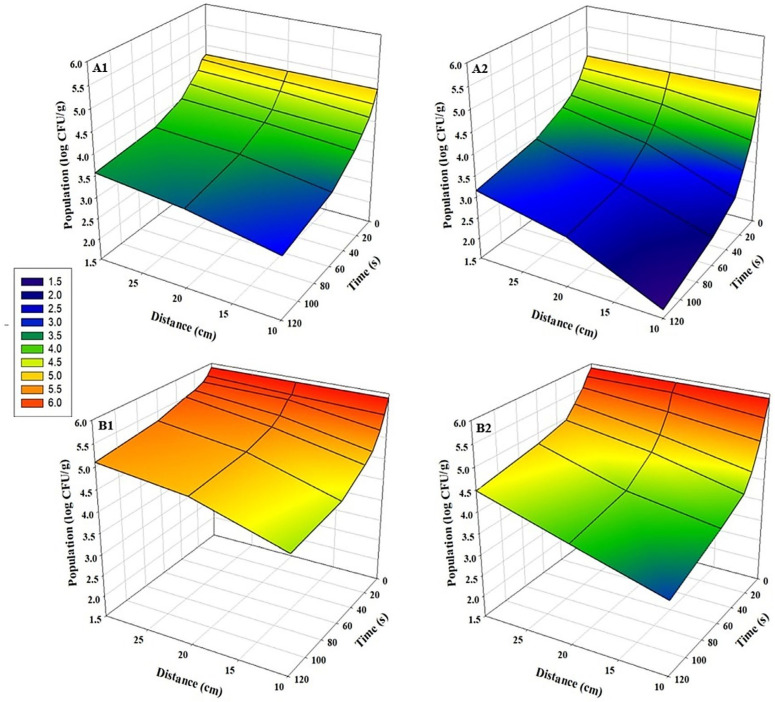
Population changes of *S. enterica* on microgreens after UV-C treatment at various time points and distances (n = 3). (**A1**) Sunflower, unidirectional treatment; (**A2**) Sunflower, bidirectional treatment; (**B1**) Radish, unidirectional treatment; and (**B2**) Radish, bidirectional treatment.

**Figure 3:**
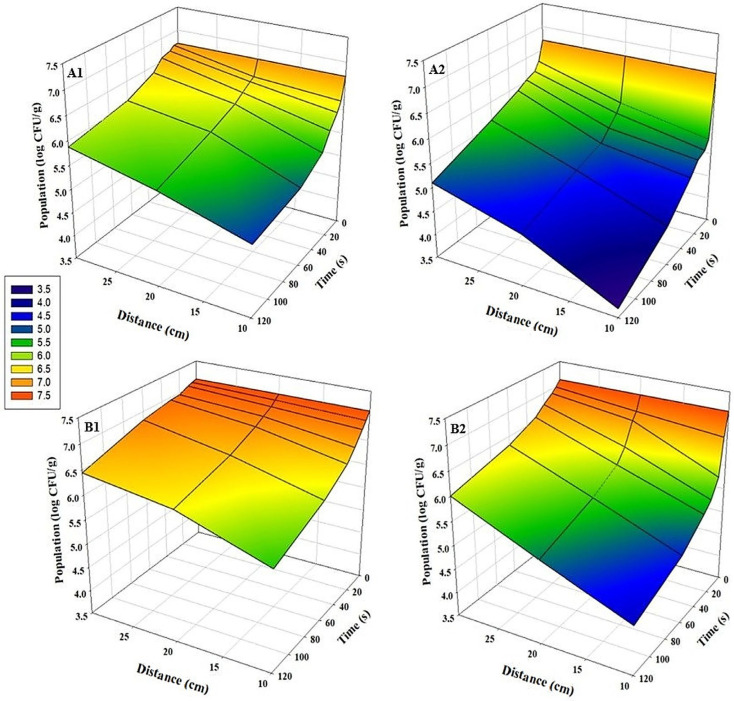
Population changes of *E. coli* O157:H7 on microgreens after UV-C treatment at various time points and distances (n = 3). (**A1**) Sunflower, unidirectional treatment; (**A2**) Sunflower, bidirectional treatment; (**B1**) Radish, unidirectional treatment; and (**B2**) Radish, bidirectional treatment.

**Figure 4:**
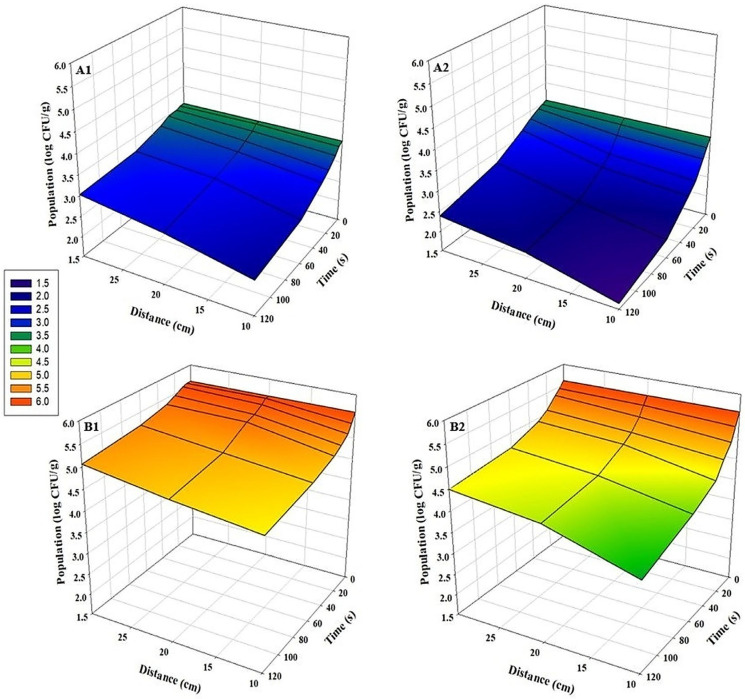
Population changes of *L. monocytogenes* on microgreens after UV-C treatment at various time points and distances (n = 3). (**A1**) Sunflower, unidirectional treatment; (**A2**) Sunflower, bidirectional treatment; (**B1**) Radish, unidirectional treatment; and (**B2**) Radish, bidirectional treatment.

For all tested pathogens, bidirectional UV-C exposure at a distance of 10 cm for 120 s resulted in the highest microbial inhibition (*p* < 0.05). A strong inverse relationship was observed between application distance and log reduction values: inhibition efficacy decreased significantly as the distance from the UV-C source increased in both sunflower and radish microgreens (*p* < 0.05) (Figure 2, Figure 3 and Figure 4). This distance-dependent trend was evident in both bidirectional and unidirectional treatments, with the greatest reductions consistently obtained at 10 cm. Across all experiments, bidirectional treatments provided significantly greater reductions than unidirectional treatments (*p* < 0.05). Slightly greater inhibition of pathogens on sunflower microgreens was observed than those on radish under identical treatment conditions. The highest *Salmonella* reductions were 3.1 log CFU/g in sunflower and 2.7 log CFU/g in radish microgreens (Figure 2(A2,B2)). For *E. coli* O157:H7, reductions reached up to 3.0 log CFU/g in sunflower microgreens and up to 2.6 log CFU/g in radish microgreens (Figure 3(A2,B2)), while for *L. monocytogenes*, reductions were up to 1.9 log CFU/g and 2.0 log CFU/g, respectively (Figure 4(A2,B2)).

### 3.3. Inhibition of TMAB and TYMC by UV-C Treatment

**Figure 5:**
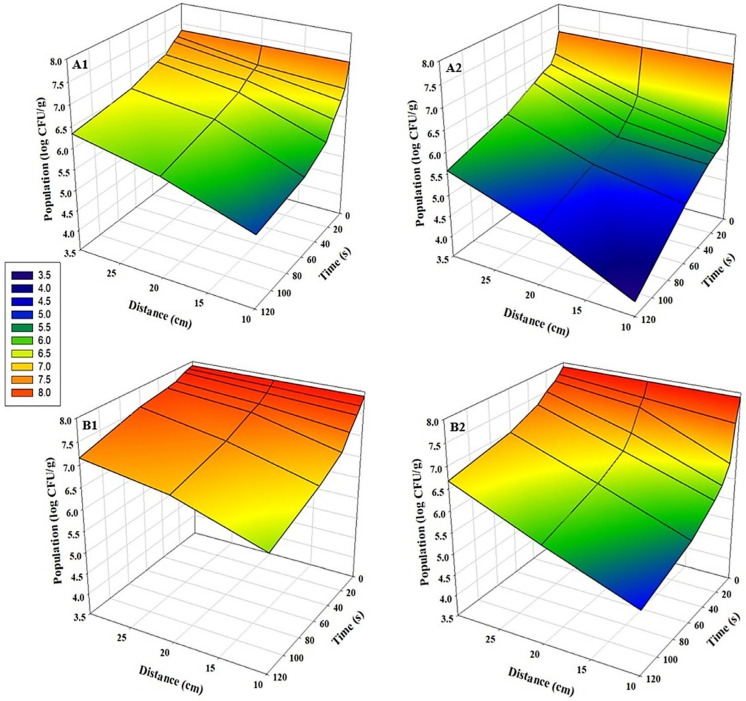
Population changes of TMAB on microgreens after UV-C treatment at various time points and distances (n = 3). (**A1**) Sunflower, unidirectional treatment; (**A2**) Sunflower, bidirectional treatment; (**B1**) Radish, unidirectional treatment; and (**B2**) Radish, bidirectional treatment.

**Figure 6:**
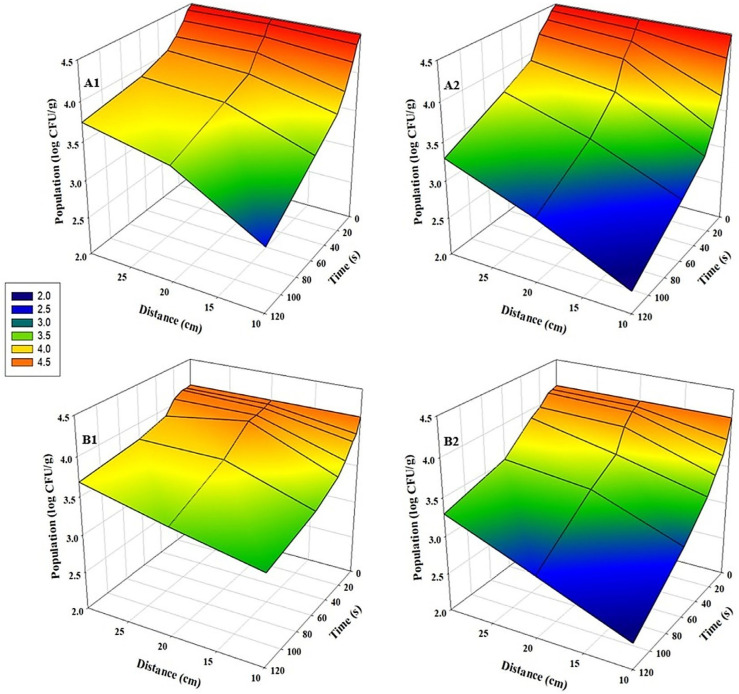
Population changes of TYMC on microgreens after UV-C treatment at various time points and distances (n = 3). (**A1**) Sunflower, unidirectional treatment; (**A2**) Sunflower, bidirectional treatment; (**B1**) Radish, unidirectional treatment; and (**B2**) Radish, bidirectional treatment.

The inactivation of TMAB and TYMC on sunflower and radish microgreens was significantly influenced by the UV-C treatment parameters. A clear inverse relationship was observed between the application distance and microbial inhibition. The efficacy of the UV-C treatment significantly decreased as the distance increased for all tested conditions (*p* < 0.05) (Figure 5 and Figure 6). The most effective reduction for both microbial groups was achieved with bidirectional UV-C treatment applied at a distance of 10 cm for 120 s. This optimal treatment resulted in a TMAB reduction up to 3.5 log CFU/g in sunflower (Figure 5(A2)) and up to 2.9 log CFU/g in radish microgreens (Figure 5(B2)). For TYMC, the maximum inhibition under the same conditions reached 2.2 log CFU/g in sunflower (Figure 6(A2)) and 1.8 log CFU/g in radish microgreens (Figure 6(B2)).

### 3.4. Storage of Microgreens After UV-C Treatment

**Figure 7:**
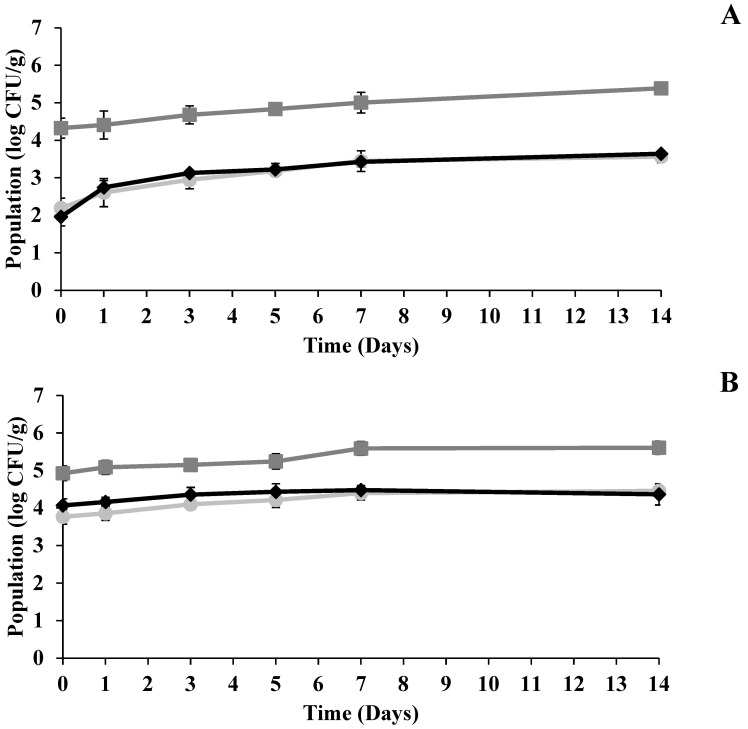
Population changes in *S. enterica* (■), *E. coli* O157:H7 (●), and *L. monocytogenes* (♦) in (**A**) sunflower and (**B**) radish microgreens during storage at 4 °C after UV-C treatment (log CFU/g) (n = 3).

**Figure 8:**
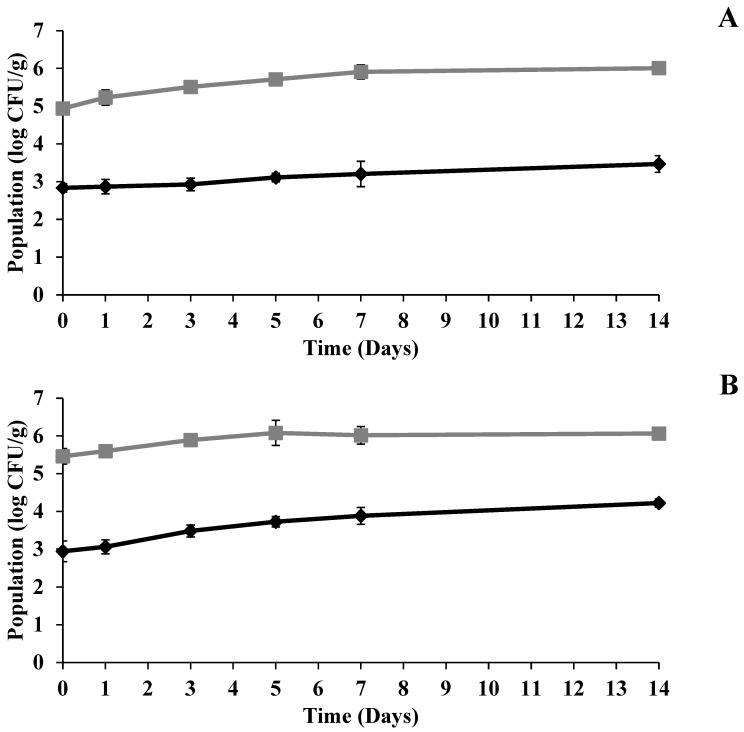
Population changes in TMAB (■), and TYMC (♦) in (**A**) sunflower and (**B**) radish microgreens during storage at 4 °C after UV-C treatment (log CFU/g) (n = 3).

The populations of the inoculated pathogens, *Salmonella*, *E. coli* O157:H7, and *L. monocytogenes*, all increased in both microgreen types over the 14-day refrigerated storage period at 4 ± 2 °C (Figure 7). In sunflower microgreens, the increases were 1.4 log CFU/g, 1.1 log CFU/g, and 1.7 log CFU/g for *S. enterica*, *E. coli* O157:H7, and *L. monocytogenes*, respectively (Figure 7A). In radish microgreens, the corresponding increases were 0.7 log CFU/g, 0.7 log CFU/g, and 0.3 log CFU/g (Figure 7B). Similarly, a significant increase (*p* < 0.05) in TMAB and TYMC populations was observed during storage in both microgreens (Figure 8). In sunflower microgreens, TMAB and TYMC populations increased by 1.1 log CFU/g and 0.6 log CFU/g, respectively (Figure 8A), after 14 days of storage. In radish microgreens, TMAB increased by 0.7 log CFU/g, while TYMC showed a higher increase of 1.3 log CFU/g by the end of the storage period (Figure 8B).

## 4. Discussion

The transfer of foodborne pathogens with large populations to the edible parts of 20 different microgreen types has been reported and comprehensively discussed with regard to contamination risks and mitigation strategies for indoor production of microgreens [6]. Contaminated seeds, agricultural water, plant growth nutrients, and soilless substrates supporting microbial survival or growth have been found to represent possible sources of pathogen transfer to microgreens. Pre- and post-harvest treatments, including the use of chemical sanitizers such as chlorine and organic acids, have been shown to have a limited reduction effect on bacterial and viral pathogen populations and microbial loads such as aerobic mesophilic bacteria [6,27,29,30,31,32,33,34]. Furthermore, treatment of microgreens followed by storage has been stated to encourage an increase in populations of foodborne pathogens and microbial loads due to possible leakage in the fragile structure of microgreen tissues during and after treatment in the same studies [29,31]. In the present study, the performance of UV-C treatment for pathogen and microbial load reduction on microgreens was assessed to overcome undesired consequences of conventional post-harvest wash mitigation strategies.

The application of UV-C treatment achieved a reduction in the population of the tested pathogens under almost all conditions to some extent. When comparing the inhibition of three pathogens transferred to sunflower and radish microgreens, *Salmonella* was identified as the most UV-C-sensitive pathogen, whereas *L. monocytogenes* exhibited the highest resistance. Similar findings were reported in a study on Kailan hybrid broccoli, where the inhibition of *E. coli*, *Salmonella*, and *L. monocytogenes* under UV-C treatment was evaluated, and *S.* Enteritidis was found to be the most sensitive while *L. monocytogenes* was the most resistant [35]. Another study showed that 1 min bidirectional UV-C exposure (1.63 kJ/m^2^) resulted in greater inhibition of *S.* Typhimurium (1.83 log CFU/g) on lettuce compared with *E. coli* and *L. monocytogenes* (1.46 and 1.44 log CFU/g, respectively) [36]. Microbial susceptibility to UV-C irradiation varies depending on numerous factors, including cell wall thickness and composition; cell size; the presence of pigmentation; the structure, size, conformation, and pyrimidine content of the genetic material; intracellular protein diversity; photoproduct formation; the physiological state of the microorganism; and the capacity to repair UV-induced damage [37,38,39]. In addition to these factors, exposure time (dose), interspecies and intraspecies (strain-level) variability, and the prevailing physiological conditions of the cell at the time of irradiation are also considered key determinants of UV-C antimicrobial efficacy [40,41]. Comparative studies conducted under identical experimental conditions have demonstrated that Gram-positive bacteria exhibit markedly greater resistance to UV-C irradiation than Gram-negative bacteria [42,43]. This difference in resistance is generally attributed to two principal mechanisms: first, the thick-peptidoglycan-layer characteristic of Gram-positive bacteria may physically limit UV-C penetration into the cell; and second, DNA repair systems in these organisms are considered to operate more efficiently [44]. Indeed, the cell wall thickness of Gram-positive bacteria ranges on average from 30 to 80 nm, whereas in Gram-negative bacteria it typically varies between 2 and 15 nm [45,46]. This structural disparity is widely regarded as one of the primary factors underlying the greater sensitivity of *Salmonella* to UV-C irradiation.

Comparable UV-C studies on various fresh produce have reported different levels of pathogen reduction depending on product surface characteristics, UV-C dose, and exposure methods. For instance, *Salmonella* reductions of 1.70–2.19 log CFU/g on tomatoes and 0.15–2.14 log CFU/g on lettuce were achieved after unidirectional UV-C treatment at doses ranging from 0.15 to 2.4 kJ/m^2^ [47]. A 1.06 log CFU/g reduction in *S.* Typhimurium was observed on alfalfa sprouts following 1 kJ/m^2^ UV-C exposure [47]. The reductions observed in the present study were greater, likely due to the bidirectional application, which increases surface contact and minimizes shadowing effects. Similarly, *E. coli* populations were reduced by 2.9 and 2.1 log CFU/g on apples and pears at 0.92 kJ/m^2^ [48], while only a 1.22 log CFU/g reduction was achieved on fresh-cut Kailan hybrid broccoli at 2.5 kJ/m^2^ [35]. Chun et al. [49] also found a 1.18 log CFU/g decrease in *E. coli* O157:H7 on ready-to-eat salad at 2 kJ/m^2^. These differences among studies can be attributed to variations in product morphology, pathogen strains, UV-C dose, and exposure method. The bidirectional UV-C treatment significantly enhances microbial inhibition in both sunflower and radish microgreens compared to unidirectional exposure. This result supports earlier reports by Kim et al. [36], who observed that bidirectional UV-C application on lettuce leaves resulted in significantly greater reductions in both *Salmonella* and *L. monocytogenes* populations compared to unidirectional exposure, with additional reductions of 0.5–1.0 log CFU/g depending on exposure time and distance.

The higher inactivation of *Salmonella*, *E. coli* O157:H7, and *L. monocytogenes* compared to longer distances in sunflower and radish microgreens at shorter UV-C distances (10 cm) in the present study is consistent with previous reports indicating that UV-C intensity decreases with increasing distance [50], leading to lower inactivation efficiency. Overall, the enhanced microbial inactivation observed under bidirectional UV-C treatment in this study supports earlier findings [51,52,53,54] that emphasize the critical role of UV-C dose, exposure geometry, and product surface characteristics in achieving effective pathogen reduction on fresh produce.

Despite similar treatment conditions, radish microgreens exhibited higher survival in the populations of the tested pathogens than those on sunflower microgreens. This variation may be explained by morphological and surface structural differences between the two species. Sunflower microgreens have broader and smoother cotyledons, which facilitate uniform UV-C penetration, whereas radish microgreens possess irregular, complex leaf surfaces that may shield microorganisms from direct exposure. Additionally, the initial bacterial load was higher in radish microgreens, further contributing to the observed differences in final pathogen counts. Previous studies demonstrated that radish microgreens grown in contaminated perlite have higher initial pathogen populations ranging from 4.15 to 6.49 log CFU/g compared with other species [6,12,27]. Such high initial pathogen populations on microgreens markedly limit the effectiveness of pathogen decontamination.

The efficacy of UV-C in reducing TMAB and TYMC populations on microgreens is dependent on similar parameters to those that are effective at reducing the tested pathogens. The superior performance of the bidirectional treatment confirms that exposing both surfaces of the microgreens to UV-C light is crucial for achieving maximum microbial inactivation. This is likely because bidirectional application ensures more uniform light distribution and minimizes shadow effects, thereby increasing the overall lethal dose delivered to the microbiota. The observed inverse relationship between application distance and microbial reduction aligns with the fundamental principle of UV light intensity, which decreases with the square of the distance from the source [55]. The significantly higher log reductions at 10 cm underscore that a higher fluence (dose) is delivered at closer proximity, leading to greater microbial damage. Under bidirectional UV-C exposure, TMAB reductions reached up to about 3.5 log CFU/g in sunflower and 2.9 log CFU/g in radish microgreens at 10 cm, while at 20–30 cm the decreases were notably lower, remaining around 2 log CFU/g. These results highlight the critical role of shorter distance in achieving effective microbial inactivation. The finding that TMAB inhibition increased with UV-C dose is consistent with the observations of Mukhopadhyay et al. [56] on tomatoes.

In bidirectional UV-C application, TYMC reductions reached approximately 2.2 log CFU/g in sunflowers and 1.9 log CFU/g in radish microgreens at 10 cm, while reductions were significantly lower at 20–30 cm, remaining around 1.6 log CFU/g. The greater resistance of TYMC compared to bacteria can be attributed to structural differences. UV inhibition occurs when radiation penetrates the cell wall, cytoplasm, and pigment layers to reach the DNA [57]. In Gram-positive bacteria, UV radiation penetration is restricted due to their thicker peptidoglycan layers, resulting in lower inactivation levels [57]. Fungi possess substantially thicker cell walls compared to the 30–80 nm peptidoglycan layer of Gram-positive bacteria, which provides an additional structural barrier and contributes to their greater resistance to UV irradiation [58]. Nonetheless, the successful reduction in TYMC confirms that UV-C is a viable technology for controlling these spoilage organisms. The dose-dependent inhibition of TYMC observed in this study is in agreement with previous research on other produce, such as tomatoes and cauliflower [56,59].

The observed sensory damage (wilted plants and damaged leaf surface) in radish microgreens from the higher UV-C dose (120 s) highlights the delicate nature of this produce and underscores the importance of optimizing treatment parameters to balance efficacy with product quality. The subsequent storage study was therefore conducted with a milder treatment (60 s) to avoid such defects. A key finding of this study is that UV-C treatment, while effective for initial decontamination, did not impart a lasting antimicrobial effect during refrigerated storage, similarly to previous studies applying pre-or post-harvest treatment to microgreens [29,31,34]. Instead, all pathogen and other microorganism populations increased in both microgreen types.

At the end of the storage period, the populations of *Salmonella*, *E. coli* O157:H7, and *L. monocytogenes* increased by 1.4–0.7, 1.1–0.7, and 1.7–0.3 log CFU/g in sunflower and radish microgreens, respectively. This increase, particularly in mesophilic pathogens such as *Salmonella* and *E. coli* O157:H7, indicates that microbial growth can continue even under refrigerated conditions, posing a potential risk in the event of post-processing contamination. Comparable results were reported by Tawema et al. [59], who observed a 2.5–3.3 log CFU/g increase in *E. coli* O157:H7 and a 2.8–3.0 log CFU/g increase in *L. monocytogenes* in UV-C treated cauliflower stored at 5 °C for 14 days. Similarly, *Salmonella* and *L. monocytogenes* populations increased during 14 days of refrigerated storage (5 °C) in UV-C treated baby spinach [60]. Consistent with the present results, previous studies have also documented increases in TMAB counts during storage of microgreens even under refrigerated conditions [29,34]. In another previous study, TMAB levels in arugula microgreens rose from 7.30 ± 0.22 to 8.80 ± 0.42 log CFU/g within ten days [61]. In addition, studies have reported increases in yeast and mold counts during storage, confirming that fungal populations can also grow under low-temperature conditions [34,61].

The increasing population of mesophilic pathogens such as *Salmonella* and *E. coli* O157:H7 under refrigerated conditions suggests a potential increase in microbiological risk in the event of post-processing contamination. The population increase during storage can be attributed to sublethal injury and mild tissue damage induced by UV-C treatment. As suggested by Tawema et al. [59] for cauliflower, such damage can release cellular nutrients, providing a more favorable environment for surviving bacteria to repair and proliferate. Our findings align with this mechanism, as the 60 s UV-C treatment likely caused minor tissue damage, facilitating microbial growth.

The significant growth of TMAB and TYMC during storage at refrigerator temperature further confirms that the treatment provided limited reduction and that the microgreens ecosystem remained conducive to microbial proliferation. Research involving the application of sanitizer washes to microgreens has reported an increase in pathogenic microorganisms after treatment, indicating that such interventions alone are insufficient to ensure long-term microbial stability [27]. The general increase in spoilage microorganisms during cold storage, even after UV-C treatment, has been previously documented in other fruits, such as mango and pineapple [62], supporting the notion that UV-C’s effect is primarily a temporary reduction rather than complete and lasting inhibition.

## 5. Conclusions

Microgreens are commonly grown in hydroponic systems ranging from major commercial greenhouses to local farms. Also, home-grown microgreens have been gaining popularity due to both their health benefits and the use of microgreens as new culinary ingredients [63]. The results of the present study showed that post-harvest UV-C application for treatment of microgreens caused a significant reduction in *Salmonella, E. coli* O157:H7 and *L. monocytogenes* populations depending on treatment time, distance and direction. The level of inhibition was also found to be influenced by the type of microgreen and the pathogen species. UV-C application is a potential method for reducing foodborne pathogen populations on microgreens in both large and small home-growing settings.

UV-C treatment can be applied during growth after harvest to reduce microbial loads and potential pathogen populations on microgreens in controlled environmental conditions, but it is important to determine the appropriate doses and conditions based on desired sensory and shelf-life expectations of microgreen species. Growers can use UV-C in their microgreen growing settings as a mitigation strategy to reduce pathogen population on crops and Zone 1 surfaces that directly contact the produce; however, the use of safe water, soilless substrate and seeds and the implementation of strict hygiene practices remain essential to ensure food safety. Although a significant decrease was observed in the pathogen population after UV-C application, the subsequent population increase during the storage process shows that UV-C application alone cannot eliminate the food safety risks associated with growing microgreens. The UV-C treatment of microgreens in combination with other food preservation methods is proposed for further studies to develop more effective mitigation strategies.

## Figures and Tables

**Table 1:** Doses of UV-C application according to different durations and distances.

Distance (cm)	Time (s)	UV-C-Dose (kj/m^2^)
10	5	0.09
	10	0.17
	20	0.34
	30	0.52
	60	1.03
	120	2.07
20	5	0.03
	10	0.07
	20	0.14
	30	0.21
	60	0.41
	120	0.83
30	5	0.03
	10	0.05
	20	0.11
	30	0.16
	60	0.32
	120	0.65

## Data Availability

The data that support the findings of this study are available from the corresponding author upon reasonable request.
